# Burnout among Emergency Nurses during COVID-19 Pandemic at Hail Governmental Hospitals in the Kingdom of Saudi Arabia

**DOI:** 10.4314/ejhs.v32i6.23

**Published:** 2022-11

**Authors:** Bahia Galal A Hassan Siam, Latifah Nawaf Alrasheedi

**Affiliations:** 1 Assistant Professor of Medical-Surgical Nursing, College of Nursing, Hail University, Saudi Arabia; 2 Master Degree in Emergency Nursing, College of Nursing, Hail University, Saudi Arabia

**Keywords:** Coronavirus, Emergency, Nurses, Pandemics, Professional burnout, Saudi Arabia

## Abstract

**Background:**

Nurses' burnout is an extended response to stressors at work, which leads to a negative self-concept and reduces the professional outcome. This study aimed to evaluate the level of burnout among emergency nurses during the COVID-19 pandemic.

**Methods:**

This cross-sectional study was conducted from October 2021 to December 2021. A total of 77 emergency nurses from King Khalid hospital 42 (54.5%), and King Salman Specialist hospital 35 (45.5%) in Hail city of Saudi Arabia, were selected using a convenient sampling method and included in the study. A self-administered questionnaire consisted of the socio-demographics data sheet, and the Copenhagen Burnout Inventory (CBI) tool to evaluate nurses' burnout.

**Results:**

The overall rate of burnout among the studied nurses was moderate, with mean scores of 39±10. The highest rate was for personal burnout with mean scores of 41.4±16.5, while the lowest score was for client-related burnout with mean scores of 37.8±10.5. The total burnout mean scores for all dimensions of CBI are higher in divorced (46.7), older age (44.5), nursing technicians (43.4), low years of experience (42.5), lower educational level (41.9), non-Saudi (41.0), and males (40.5).

**Conclusion:**

The prevalence of burnout among nurses in government hospitals in Hail city was moderate, which should be considered. No statistically significant association were found between socio-demographic variables and the mean scores of CBI. Therefore, it is necessary to investigate the factors associated with the occurrence of burnout, provide a training program to reduce it, and improve the mental and physical health of the emergency nurses.

## Introduction

The Coronavirus disease 2019 (COVID-19), the global pandemic causing Severe Acute Respiratory Syndrome Corona virus-2 (SARS CoV-2), has devastated the world resulting in 215,569,910 infections and 4,490,024 deaths until Late August 2021 (1). The emergence of COVID-19 and its enervating effects on lifestyle and economies around the world is very disturbing. In addition, COVID-19 pandemic has affected all sectors directly or indirectly, and the crisis is worse on the already overburdened health systems in many countries, as well as healthcare workers (HCWs), especially nurses (2, 3). This incorporate affects the psychological status of all healthcare workers, mainly the nurses who have direct contact with those patients and work with them frequently and closely. Nurses were those who assigned and handled the visual triage point at every entry in every hospital meanwhile maintaining all standards of social distancing protocols as directed by Saudi Ministry of Health (MOH), becoming more exposed and in danger of the disease (4). Thus, they become overwhelmed by fear for the security of their own health, their close relations, and their patients and eventually burnout (5).

Burnout is a psychological syndrome emerging as a prolonged response to chronic interpersonal stressors on the job (5, 6). The concept of burnout in health care emerged in the late 1960s as a way to describe the emotional and psychological stress experienced by clinic staff caring for structurally vulnerable patients in free clinics (7, 8). The Copenhagen Burnout Inventory (CBI) is a questionnaire with three sub-dimensions: Personal burnout, work-related burnout, and client-related burnout. Personal burnout to compares individuals regardless of occupational status. Work-related and client-related burnout is the degree of physical and psychological fatigue and exhaustion that is perceived by the person as related to his/her work and work with clients (9).

During the COVID-19 pandemic, the emergency nurses were the first health care professionals to treat patients infected (10). The workload of emergency nurses is quite heavy; the emergency room staff are under sustained stress owing to the crowded working environment, the severity of cases, furthermore, unsafe working environments, and critical care decisions (11,12). Furthermore, nurses' burnout is a frequent and serious problem that can cause many serious negative health implications not only for themselves but also for patients to whom they provide care, their colleagues, and the healthcare facilities that they belong. More burnout symptoms mean lower productivity levels, exposing nurses to multiple errors in their clinical settings, and negligence in handling patients (13). Therefore, evaluating the prevalence of burnout symptoms of the COVID-19 pandemic among healthcare workers, particularly nurses, is very crucial in writing policies and interventions to maintain their psychological well-being and safety. To the best of our knowledge, in Saudi Arabia, few research studies investigated the prevalence of burnout of COVID-19 among nurses in the Hail region. This study aimed to evaluate the level of burnout among emergency nurses during the COVID-19 pandemic at Hail Governmental Hospitals in Saudi Arabia.

## Methods

**Study design and setting**: A cross-sectional descriptive research design was utilized in this study. The study was conducted at the emergency departments of King Khalid Hospital and King Salman Specialist Hospital in Hail city of Saudi Arabia.

**Study population**: The current study included all available nurses, both genders, and any nationality who worked at the Emergency Departments of King Khalid Hospital and King Salman Specialist Hospital in Hail city, Saudi Arabia.

**Sample size determination**: The total number of nurses working at the Emergency Departments of King Khalid Hospital and King Salman Specialist Hospital in Hail city of Saudi Arabia was 96 nurses. All nurses with minimum one-year working experience were included in the present study. At the end, a total of 77 nurses, both genders, aged 20 to 60 years, selected by a convenient sampling method, with a response rate 80.20% were included in the current study.

Study period: The study was conducted within three months (from the beginning of October 2021 to the end of December 2021).

**Data collection**: A self-administered questionnaire consisted of two parts: Part I: The Nurses' socio-demographic data sheet including age, gender, nationality, marital status, education level, marital status, job title, years of experience, and the work setting (hospital); and Part II: The Copenhagen Burnout Inventory (CBI): A 5-point Likert scale developed and validated by Kristensen et al., 2005 was used to evaluate nurses' burnout. This inventory is highly valid and reliable, as it has been applied in different domains in various studies and shows high internal validity and reliability (9).

The Copenhagen Burnout Inventory (CBI): The CBI consists of 19 items comprising three subscales: personal burnout (six items), work-related burnout (seven items), and client-related burnout (six items). Twelve items have responses of frequency along a 5-point Likert scale ranging from zero (never), 25 (seldom), 50 (sometimes), 75 (often), and 100 (always). Seven items using response categories according to intensity (the first three questions of work-related burnout and the first four questions of client-related burnout) scores ranged from zero (very low degree) to 100 (very high degree). The Last question, “Q-19”, is negatively worded that has been revised before data analysis (9).

In the current study, the mean scores were calculated and presented per each item; then, the average total mean scores were presented for each subscale and for the overall scores of CBI. The total score is the average of the scores on the items. The total score is classified into three categories; mild burnout (<50%), moderate burnout (50–75), and high (75.1–99), a score (100%) considered severe burnout (9). Separate total scores were reported for each sub-dimension, and the total score of the test is the average score of all three sub-dimensions. Additionally, pilot study was carried out on twenty participants to enable the researcher to examine the tools of the study.

**Statistical analysis**: The Statistical Package for the Social Sciences (SPSS) version 28.0 software was used for all statistical analyses. All categorical variables were presented as frequencies and percentages, while continuous variables were presented as means and standards of deviation. Then the prevalence of burnout was calculated using a binomial “exact” calculation. P-values that are <0.05 was considered significant.

**Ethical approval:** Ethical approval was obtained from the Ethics Committee for Bioethics Research in Hail Health (No. 2021/45), as well as approval letter was obtained from Hail hospitals. Additionally, a questionnaire with a statement of consent on the front page was obtained from each participant without force prior to the study after reading a statement that will provide a full explanation of the study's purpose. Also, the protection of the privacy and confidentiality of the research participants was ensured.

## Results

Table 1 shows that 42 (54.5%) of the included emergency nurses were from King Khalid hospital, and 35 (45.5%) were from King Salman Specialist hospital. 55.8% of the emergency nurses were in the age group from 20 to less than 35 years, 64.9% of them were females, 51.9% were non-Saudi citizens, and 64.9% were married. As regards educational levels and years of experience, 80.5% and 53.2% of nurses had bachelor degree in nursing and 5 to 10 years of experience, respectively.

**Table 1:** Socio-demographic characteristics of the emergency nurses

Variables	Frequency (n=77)	Percent
**Hospital name**		
King Khalid hospital	42	54.5
King Salman Specialist hospital	35	45.5
**Age (years)**		
20 to < 35	43	55.8
35 to < 45	22	28.6
≥ 45 years	12	15.6
**Gender**		
Male	27	35.1
Female	50	64.9
**Nationality**		
Saudi	37	48.1
Non-Saudi	40	51.9
**Marital status**		
Single	24	31.2
Married	50	64.9
Divorced	2	2.6
Widowed	1	1.3
**Education level**		
Diploma in nursing	9	11.7
Bachelor degree in nursing	62	80.5
Master degree in nursing	6	7.8
**Profession**		
Nursing technician	39	50.6
Nursing specialist	21	27.3
Senior nursing specialist	11	14.3
Consultant	6	7.8
**Years of experience**		
Less than 5 years	22	28.6
From 5 to 10 years	41	53.2
More than 10 years	14	18.2

Data are expressed as percentage for categorical variables.

Table 2 shows the mean scores of personal burnouts of the CBI among emergency nurses. The results revealed that the mean scores of personal burnouts range from 36.4 to 47.1, with a total average mean score of 41.4.

**Table 2:** Mean scores of personal burnout of the CBI among emergency nurses

Personal burnout		Frequency (n=77); percentage of burnout (%)					Mean scores
		
		Always	Often	Some-times	Seldom	Never	
1	How often do you feel tired?	7 (9.1)	12 (15.6)	19 (24.7)	20 (26.0)	19 (24.7)	39.6
2	How often are you physically exhausted?	6 (7.8)	13 (16.9)	20 (26.0)	22 (28.6)	16 (20.8)	40.6
3	How often are you emotionally exhausted?	8 (10.4)	15 (19.5)	14 (18.2)	19 (24.7)	21 (27.3)	40.3
4	How often do you think: “I can't take it anymore”?	6 (7.8)	9 (11.7)	17 (22.1)	27 (35.1)	18 (23.4)	36.4
5	How often do you feel worn out?	9 (11.7)	17 (22.1)	16 (20.8)	18 (23.4)	17 (22.1)	44.5
6	How often do you feel weak and susceptible to illness?	10 (13.0)	20 (26.0)	10 (13.0)	25 (32.5)	12 (15.6)	47.1
**Overall personal burnout: Mean±SD = 41.4 ± 16.5**							

Data are expressed as percentage for categorical variables. The prevalence of burnout was calculated using a binomial “exact” calculation. CBI: Copenhagen Burnout Inventory.

Table 3 shows mean scores of work-related burnouts of the CBI among emergency nurses. The results demonstrated that the mean scores of work-related burnouts range from 31.8 to 46.8 with a total average mean score of 39.8.

**Table 3:** Mean scores of work-related burnout of the CBI among emergency nurses

Work-related burnout assessment		Frequency (n=77); percentage of burnout (%)					Mean scores
		
		Always	Often	Some-times	Seldom	Never	
1	Is your work emotionally exhausting?	5 (6.5)	21 (27.3)	21 (27.3)	19 (24.7)	11 (14.3)	46.8
2	Do you feel burnt out because of your work?	4 (5.2)	11 (14.3)	14 (18.2)	28 (36.4)	20 (26)	34.1
3	Does your work frustrate you?	8 (10.4)	15 (19.5)	16 (20.8)	26 (33.8)	12 (15.6)	43.8
4	Do you feel worn out at the end of the working day?	9 (11.7)	13 (16.9)	19 (24.7)	19 (24.7)	17 (22.1)	42.9
5	Are you exhausted in the morning at the thought of another day at work?	10 (13.0)	7 (9.1)	24 (31.2)	21 (27.3)	15 (19.5)	42.2
6	Do you feel that every working hour is tiring for you?	10 (13.0)	19 (24.7)	18 (23.4)	17 (22.1)	13 (16.9)	31.8
7	Do you have enough energy for family and friends during leisure time?	8 (10.4)	11 (14.3)	24 (31.2)	22 (28.6)	12 (15.6)	36.7
**Overall work-related burnout: Mean±SD = 39.8 ± 11.3**							

Data are expressed as percentage for categorical variables. The prevalence of burnout was calculated using a binomial “exact” calculation. CBI: Copenhagen Burnout Inventory.

Table 4 shows mean scores of client-related burnouts of the CBI among emergency nurses. The results showed that the mean scores of client-related burnouts range from 28.6 to 49.7, with a total average mean score of 37.8.

**Table 4:** Mean scores of client-related burnout of the CBI among emergency nurses

Client-related burnout assessment		Frequency (n=77); percentage of burnout (%)					Mean scores
		
		Always	Often	Some-times	Seldom	Never	
1	Do you find it hard to work with clients?	10(13.0)	12 (15.6)	30 (39.0)	15 (19.5)	10 (13.0)	36.7
2	Do you find it frustrating to work with clients?	6 (7.8)	11 (14.3)	23 (29.9)	26 (33.8)	11 (14.3)	36.7
3	Does it drain your energy to work with clients?	5 (6.5)	23 (29.9)	17 (22.1)	18 (23.4)	14 (18.2)	28.6
4	Do you feel that you give more than you get back when you work with clients?	9 (11.7)	18 (23.4)	15 (19.5)	27 (35.1)	8 (10.4)	39.3
5	Are you tired of working with clients?	9 (11.7)	7 (9.1%)	21 (27.3)	21 (27.3)	19 (24.7)	36.4
6	Do you sometimes wonder how long you will be able to continue working with clients?	6 (7.8)	15 (19.5)	23 (29.9)	18 (23.4)	15 (19.5)	49.7
**Overall client-related burnout: Mean±SD = 37.8 ± 10.5**							

Data are expressed as percentage for categorical variables. The prevalence of burnout was calculated using a binomial “exact” calculation. CBI: Copenhagen Burnout Inventory.

**Figure 1:**
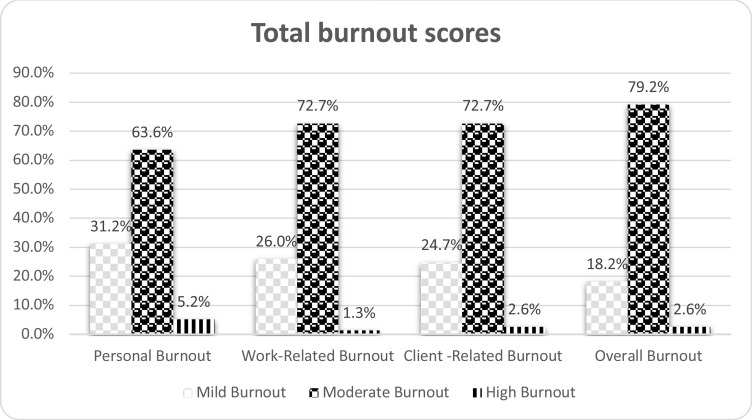
The percentage of total scores and sub-dimensions of Copenhagen Burnout Inventory

Figure 1. shows that moderate burnout represents the highest total burnout scores of CBI and total sub-dimensions in terms of personal, work-related, and client-related burnout with percent 79.2%, 72.7%, 72.2%, and 63.6%, respectively. Table 5 reveals that the total burnout mean scores for all dimensions of CBI are higher in divorced (46.7), older age (44.5), nursing technicians (43.4), low years of experience (42.5), lower educational level (41.9), King Khalid hospital (41.6), non-Saudi (41.0), and males (40.5). However, the work-related burnout mean scores are higher in King Khalid hospital, young ages, single, and female nurses with an overall non-significant statistical difference (p values > 0.05 for all).

**Table 5:** Relationship between socio-demographic characteristics of the emergency nurses and the mean scores of CBI

Items	Burnout mean scores (n=77)							
	
	Personal burnout		Work-related burnout		Client-related burnout		Total burnout	
	
	Mean	P-value	Mean	P-value	Mean	P-value	Mean	P-value
**Hospital name**								
King Khalid	41.8	0.387	44.2	0.216	39.7	0.514	41.6	0.627
King Salman Specialist	39.1		38.5		32.4		36.7	
**Age (years)**								
20 to < 35	38.1	0.062	39.0	0.115	38.4	0.813	38.6	0.127
35to < 45	42.9		37.8		36.9		39.2	
≥ 45 years	50.3		45.8		37.2		44.5	
**Gender**								
Male	44.1	0.286	40.7	0.574	37.3	0.745	40.5	0.453
Female	39.9		39.2		38.7		39.1	
**Nationality**								
Saudi	39.0	0.214	38.3	0.287	37.5	0.762	38.3	0.189
Non-Saudi	43.6		41.1		38.2		41.0	
**Marital status**								
Single	45.7	0.316	42.11	0.096	39.6	0.540	42.4	0.172
Married	39.0		37.9		37.5		38.1	
Divorced	52.1		55.4		31.3		46.7	
Widowed	37.5		42.8		29.2		36.8	
**Education**								
Diploma in nursing	47.2	0.461	44.4	0.415	40.3	0.420	41.9	0.665
Bachelor degree in	40.3		39.2		38.2		39.2	
nursing	45.4		38.7		33.8		41.4	
Master degree in nursing								
**Profession**								
Nursing technician	47.8	0.237	45.1	0.192	39.0	0.577	43.4	0.437
Nursing specialist	41.8		40.8		37.7		39.7	
Senior nursing specialist	36.1		38.6		37.1		38.3	
Consultant	45.8		33.9		32.6		37.3	
**Years of experience**								

Less than 5 years	42.0	0.200	43.4	0.385	38.8	0.661	42.5	0.446
From 5 to 10 years	38.8		38.1		37.3		39.0	
More than 10 years	41.4		39.4		36.0		39.1	

The prevalence of burnout was calculated using a binomial “exact” calculation. P value less than 0.05 was considered as statistically significant. CBI: Copenhagen Burnout Inventory.

## Discussion

The COVID-19 pandemic caused an overall rise in psychological problems, including anxiety, depressive disorders, and burnout among health care workers (14). The emergency nurses are the first health care professionals to treat patients infected during the COVID-19 pandemic. The workload of emergency nurses is quite heavy because, generally, patients who are rushed to the emergency departments are emergency patients who need to get health services as quickly and accurately as possible (15). This study aimed to assess the level of burnout among emergency nurses during the COVID-19 pandemic at Hail Governmental Hospitals in Saudi Arabia.

Regarding socio-demographic data of the studied nurses; more than half of them were within the age group 20 to 35 years; this may be due to the middle age is required for the nature of work in emergency departments that requires quick, immediate responses, especially during the pandemic of COVID 19. Around two-thirds were females, married, and non-Saudi. As regards the educational level, most of them had bachelor degree in nursing. More than half of our subjects work as nursing technicians with 5 to 10 years of work experience. These findings are similar to a previous study, which found that the minority of nurses were Saudis, more than half of them were bachelor's degree holders, and more than half of nurses have more than six years of experience (16). In contrast, other studies stated that younger age, higher educational level, and higher degrees increased nurses' burnout (10, 17).

As regards nurses' burnout, the results of this study represented that the highest mean scores of CBI sub-dimensions were for personal burnout (41.4) and work-related burnout (39.7), while client-related burnout represented the lowest means (37.8). These findings are supported by a study published in 2021 that reported higher personal and work-related burnout levels (19). In addition, another study stated that more than half of the respondents had personal burnout, and more than one-third had work-related and client-related burnout (20). However, in other study the researcher found that the study subjects had lower burnout means that ranged from 9.8 to 34.58 (15).

The total burnout scores had classified and presented in percentage based on the scoring system of CBI into mild, moderate, and high (9). The moderate scores represented the highest percentage of overall total scores and sub-dimensions (personal, work-related, and client-related burnout). These results are in contrast with a previous study conducted in Saudi Arabia that showed a high level of burnout among HCWs (21). Worldwide, a systematic review of results showed a significant prevalence of nurses' burnout during the COVID-19 pandemic in 16 studies in different countries (22). From our point of view, the moderate prevalence of burnout among nurses in Hail governmental hospitals may be related to nurses' coping after two years of pandemic and the efforts of the MOH in Saudi Arabia during the pandemic. In all phases of the pandemic, the ministry supports the HCWs in overcoming any difficulties. In spite of that, scores will be considerable.

The results of the current study revealed that; there was no significant statistical relationship between the socio-demographic characteristics of the emergency nurses and the mean scores of CBI (P values > 0.05 for all). This finding goes in the same line with previous studies, which concluded that there was no association between burnout and socio-demographic factors such as age, gender, education level, marital status, and work position (15, 23). However, the results revealed that all burnout mean scores in all sub-dimensions of CBI were higher in old ages, males, non-Saudi, divorced, with lower educational levels, nursing technicians, and low years of experience. Except; the work-related burnout mean scores were higher among young ages, single and female nurses, with no significant statistical difference. In relation to age, these results are the same as the results of a study conducted in Egypt 2020 that reported that older nurses are more likely to develop burnout syndrome rather than younger nurses (24). However, this finding is in contrast with a study conducted at Saudi Arabia in 2021, that found younger HCWs who were aged between 22 and 35 years were more likely to experience burnout than older HCWs (23). The researchers believe that increasing work-related burnout in the age group 25–35 may be due to a lack of enough experience in dealing with pandemics.

Previous research stated that gender is significantly associated with burnout. Moreover, gender is a controversial issue since our review showed that females have higher levels of emotional exhaustion, but males have higher levels of depersonalization (24). Regarding nationality, the burnout scores in all domains were higher in non-Saudi nurses; this finding reflects the negative effect of being away from home and family on overall support. Concerning marital status, the results of the current study stated that the rate of burnout was higher among married nurses, this can be explained by the fact that married nurses may be more anxious than others because of their fear of transmitting the infection to their families and relatives. Other studies represented that marital status is significantly associated with burnout (24,25,26). For the educational level and nurses' profession, the results of the current study reveal that the burnout rate was higher in nursing technicians and nurses who had diplomas; this may be explained by a lack of academic qualifications and job rank compared to specialists and nursing consultants, according to years of experience. The main strength of our study was its being the first study, which shows the prevalence of burnout among emergency nurses during COVID-19 pandemic at Hail governmental hospitals in the Kingdom of Saudi Arabia. The main limitations of this study are its cross-sectional design; the causal relationship could not be determined, and it limits the generalizability of our results. In addition, a self-administered questionnaire and its small sample size are other limitations.

In conclusion, the level of burnout among the studied nurses during the COVID-19 pandemic was moderate. The highest rates were for personal burnout; the lowest scores for client-related burnout. There was no significant statistical relation between the socio-demographic characteristics of the emergency nurses and the mean scores of CBI. The outcome of this research provides basic information geared toward contributing to programs and interventions were for the reduction of burnout in nurses in Saudi governmental hospitals, especially during the pandemic. Nurses need support from family and society. Furthermore, investments in mental well-being strategies and psychological interventions are encouraged to enhance the health of nurses during possible future pandemics. Further future studies with large sample size are required to confirm these findings.
